# Complete heart block in patients infected with SARS-CoV-2: A case series from a developing country

**DOI:** 10.1016/j.amsu.2021.102828

**Published:** 2021-09-06

**Authors:** Muhammad Hammad Sharif, Abdul Wali Khan, Madeeha Khaleeque, Ammer Haffar, Vikash Jaiswal, David Song, Mohamed Abdelghffar, Saad Ahmad, Talal Almas, Muhammad Hanif

**Affiliations:** a Cardiology Hayatabad Medical Complex, Pakistan; b IM Hayatabad Medical Complex, Pakistan; c Internal Medicine Khyber Teaching Hospital, Pakistan; d Jersey City Medical Center, New Jersey, USA; e Larkin Community Hospital, South Miami, Fl, USA; f Icahn School of Medicine at Mount Sinai, NY, USA; g Royal College of Surgeons in Ireland, Dublin, Ireland; h Hayatabad Medical Complex Peshawar, KPK, Pakistan

**Keywords:** Complete heart block, SARS-CoV-2, COVID-19, Novel coronavirus

## Abstract

Coronavirus Disease 19 (COVID-19) has led to a global pandemic and has been the center of attention across the entire medical community. This novel virus was initially thought to affect primarily the respiratory system, but now it is evident that it has a multitude of effects on the human body. Our point of interest is to establish the effect of COVID-19 infection on the conducting system of the heart. We present a case series of four patients who developed complete heart block (CHB) shortly after being infected with COVID-19 without any previous known risk factors of complete heart block. There have only been a few previous case reports on the occurrence of CHB in COVID-19 patients highlighting the importance and the need of our case series to the literature of cardiovascular outcomes in COVID-19 patients. Our case series highlight that COVID-19 can indeed affect the conduction system of the heart and cause CHB in patients who then recovered spontaneously further elucidating the transient nature of cardiovascular effects caused by the novel virus.

## Highlights

• COVID-19 was initially thought to affect primarily the respiratory system, but now it is evident that it has a multitude of effects on the human body including the conduction system of the heart that can cause complete heart block (CHB).
• Other etiologies of CHB must be ruled out including ischemic heart disease, hyperkalemia, hypothyroidism, infections and rate limiting drugs affecting the AV node conduction including BB, CCB, and digoxin.
• There are many potential effects of COVID-19 and CHB should be considered and close monitoring with telemetry will be beneficial.

## 1 Introduction

In December 2019 a novel coronavirus, SARS-CoV-2, was initially identified in the city of Wuhan, Hubei province of China as the pathogen causing coronavirus disease 2019 (COVID-19) [1]. The disease continued to spread globally and by the end of January 2020, SARS-CoV-2 was declared as a public health emergency of international concern [1]. COVID-19 predominantly affects the respiratory system, and most commonly presents with signs and symptoms of pneumonia. However, there have been cases where COVID-19 affected the cardiovascular system as well. In fact, it is estimated that up to 19.7% of patients were noted to have a cardiac injury based on the literature from Wuhan, China [2,3]. There are a variety of mechanisms by which the COVID-19 can affect the cardiovascular system including endothelial dysfunction, diffuse microangiopathy with thrombosis and increased angiotensin II levels, and hyperinflammation of the myocardium which can result in acute coronary syndrome (ACS), myocarditis, heart failure (HF), cardiac arrhythmias and sudden death [6].

Our primary interest is complete heart block (CHB) in the setting of COVID-19. The prevalence of CHB in a general population appears to be 0.02–0.04% [4]. There are only a few case reports on the effects of COVID-19 on the conduction system of the heart [5]. COVID-19 induced heart block cases present the same as other cases of CHB with clinical features of shortness of breath, bradycardia, dizziness, and syncopal episodes. It can be diagnosed by 24 h telemetry monitoring and electrocardiogram (ECG). Management includes treating the underlying etiology; however, if a patient becomes symptomatic then deployment of a temporary pacemaker can be implemented to alleviate the symptoms. If the cardiac insult becomes permanent, a permanent pacemaker can be used. We highlight a case series of four patients with COVID-19 who developed CHB with no other risk factors or underlying etiology that could be attributed to a high degree atrioventricular (AV) block. This case series emphasizes the need to monitor patients with COVID-19 for other complications such as arrhythmias in addition to the typical respiratory complications. All our work has been reported in line with the PROCESS criteria and guidelines [7].

## 2 Case presentation

### 2.1 Case #1

Patient is a 56 years old male Pakistan native, with a past medical history of diabetes (DM) who presented with complaints of fever and shortness of breath for the last seven days. On exam, he was febrile to 102.6 °F and had an oxygen saturation of 79% on room air (RA). He appeared to be very tachypneic to a respiratory rate (RR) of 27 and appeared to be in respiratory distress. Chest auscultation revealed bilateral coarse crackles. On admission, lab results were significant for lymphopenia and elevated inflammatory markers including C-reactive protein (CRP) of 87 mg/dL (normal: less than 10 mg/dL), D-dimer of 810 ng/mL (normal: less than 250 ng/mL), Ferritin 1245 ng/mL (normal: 24–336 ng/mL), and LDH to 564 U/L (normal: 140–280 U/L).

Furthermore, chest radiography revealed widespread peripheral opacities. Initial ECG showed normal sinus rhythm at a rate of 82 beats per minute (bpm), normal intervals, normal axis, and no ST or T wave changes. A nasopharyngeal swab test for COVID-19 on real-time reverse transcriptase polymerase chain reaction (PCR) assay was positive.

**Fig. 1:**
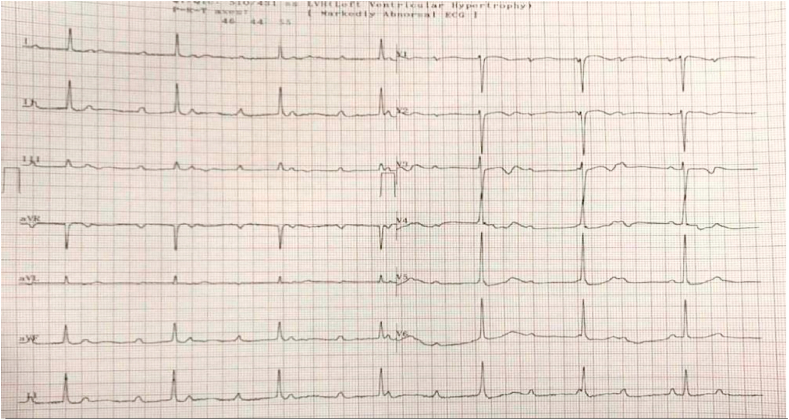
This is the electrocardiogram (ECG) of patient no 1 on his 3rd day of admission showing AV disassociation with independent atrial and ventricular activity consistent with complete heart block.

Patient was subsequently started on supplemental O_2_, intravenous (IV) Ceftriaxone 2 gm twice daily (BID), Azithromycin 500 mg BID, IV Clexane (1 mg/kg) BID, IV Methylprednisolone 1 g (05-2 mg/kg), Paracetamol 500 mg tablets three time a day (TID), zinc and multivitamin supplements. On the third day of admission, he was complaining of dizziness and one episode of syncope. His cardiac telemetry showed a pattern of CHB with a rate of 36 bpm (Fig. 1). Further work-up performed to evaluate for other etiologies of CHB were unremarkable including a normal thyroid stimulating hormone (TSH), serial negative troponin, negative orthostatic hypotension, and left ventricular ejection fraction of 70% with no segmental wall motion abnormality noted on transthoracic echocardiogram (TTE). Patient did not complain of any episode of chest pain and his serum potassium was 4.7 (normal: 3.5–5 mEq/L). Further review of the medications revealed absence of AV nodal blocking agents including beta blockers (BB), calcium channel blockers (CCB), and digoxin. Given his persistent symptoms, a temporary pacemaker (TPM) was placed.

By the ninth day of hospitalization his dizziness had improved, telemetry showed that he was in normal sinus rhythm, and subsequently his TPM was removed. Patient overall became asymptomatic, and recovered from COVID-19 with no residual complications. He was discharged and on routine follow ups, he remained asymptomatic and repeated ECG showed normal sinus rhythm with no evidence of CHB.

### 2.2 Case #2

Patient is a 48 year old male with a past medical history of hypertension (HTN) who presents with complaints of fever as high as 103.6 F, myalgias, non-bloody diarrhea and shortness of breath. On examination he appeared very lethargic, had increased work of breathing, and was found to have an oxygen saturation of 75% on RA. Chest auscultation revealed bilateral coarse crackles. Chest radiography revealed bilateral peripheral opacities. Labs were notable for the following elevated inflammatory markers: CRP 87 mg/dL, D-dimer 765 ng/mL, Ferritin 1245 ng/mL, and LDH 986 U/L, and his COVID-19 PCR was positive. ECG on presentation showed normal sinus rhythm at a rate of 73 bpm, normal intervals, normal axis, and T wave inversion noted on V_1_–V_4_. Serial troponins were within normal limits and the patient did not complain of any chest pain or palpitation.

**Fig. 2:**
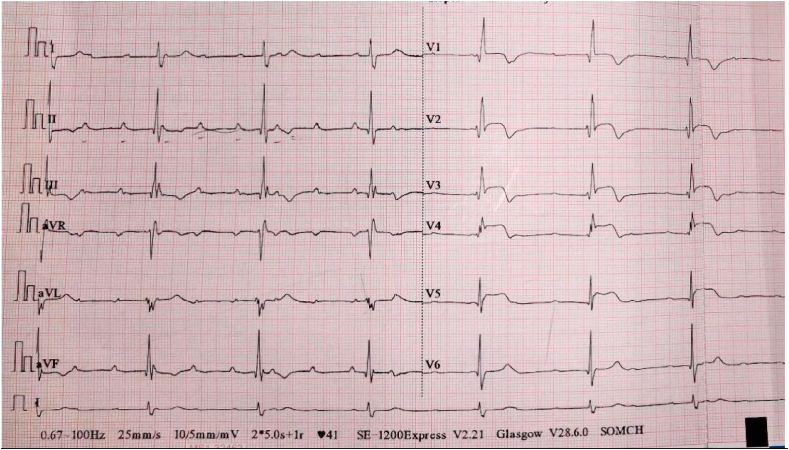
This is the electrocardiogram (ECG) of patient no 2 on his 5th day of admission showing complete heart block as evidenced by independent atrial and ventricular activity.

Patient was subsequently treated with supplemental O_2_, IV Ceftriaxone 2 gm BID, Azithromycin 500 mg tablets BID, IV Clexane (1 mg/kg) BID, IV Methylprednisolone 1 g (05-2 mg/kg), Paracetamol 500 mg tablets TID, zinc and multivitamin supplements. On his fifth day of admission, his telemetry demonstrated a CHB and repeated ECG also consistent with CHB (Fig. 2). He had no prior history of AV blocks. Medication reconciliation was unremarkable for any AV nodal blocking agents. Serum potassium, thyroid function tests, serial troponins, and TTE were all within normal limits. Patient was otherwise asymptomatic.

During his hospital course he was monitored with no other intervention over 8 days, his telemetry subsequently showed normal sinus rhythm as he recovered from COVID and was discharged with a plan for outpatient follow-up. On routine follow up, repeat ECG showed no signs of CHB.

### 2.3 Case #3

Patient is a 57 year old female with no past medical history who presented with complaints of high grade fever and sore throat for the last 7 days. Her initial vitals showed that she was febrile to 104.6 F and was hypoxic to 85% on RA. On exam, she was ill appearing, diaphoretic and in respiratory distress, chest auscultation showed diffuse coarse crackles. Lab findings were remarkable for significantly elevated inflammatory markers including CRP of 106 mg/dL, D dimer of 1098 ng/mL, Ferritin of 943 ng/mL, and LDH of 861 U/L. Chest radiography showed bilateral peripheral lung opacities and her COVID-19 PCR resulted positive. ECG on presentation showed normal sinus rhythm at a rate of 79 bpm, normal intervals, normal axis without ST or T wave changes.

**Fig. 3:**
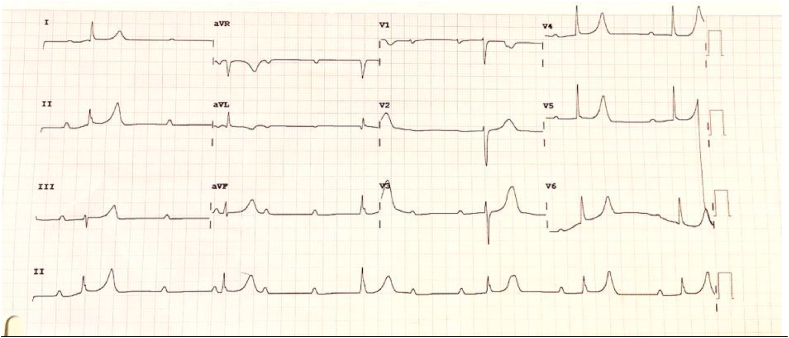
This is the electrocardiogram (ECG) of patient no 3 on her 5th day of admission showing complete heart block with feature of total AV dissociation.

Patient was started on supplemental O_2_, IV Ceftriaxone 2 gm BID, Azithromycin 500 mg tablets BID, IV Clexane (1 mg/kg) BID, IV Methylprednisolone 1 g (05-2 mg/kg), Paracetamol 500 mg tablets TID, zinc and multivitamin supplements. Her hospital course was complicated by bradycardia to 50 bpm and spells of dizziness. ECG done at the time was revealing for a CHB (Fig. 3). Further chart review and history revealed that the patient never had a prior abnormal ECG, her serial troponins were negative, potassium, thyroid function tests or other electrolytes were all within normal limits. In addition, TTE was unremarkable. She was never on any AV nodal blocking agents.

As she was symptomatic, the decision was made to place a TPM and to continue monitoring the patient. Five days later, ECG and telemetry revealed \normal sinus rhythm and the TPM was removed. Patient recovered from her COVID-19, no longer complained of dizziness and was subsequently discharged. During her follow up, the repeat ECG showed normal sinus rhythm with no evidence of AV block.

### 2.4 Case #4

Our last patient is a 42 year old female with a past medical history of DM who presented with complaints of high grade fever, shortness of breath, sore throat and severe body aches. Her exam was significant for a fever of 103.6 F, oxygen saturation of 78% on RA. She was diaphoretic and short of breath. Diffuse crackles throughout lung fields were heard on auscultation. Lab findings showed that she had elevated inflammatory markers including CRP of 87 mg/dL, D-dimer 917 ng/mL, Ferritin of 1245 ng/mL, and LDH of 598 U/L and a positive COVID-19 PCR. Chest radiography revealed peripheral ground glass opacities. Her ECG on admission showed normal sinus rhythm at a rate of 79 bpm, normal intervals, normal axis with T wave inversion noted on.

#### 2.4.1 Lead 1, aV_L_ and V_2_

**Fig. 4:**
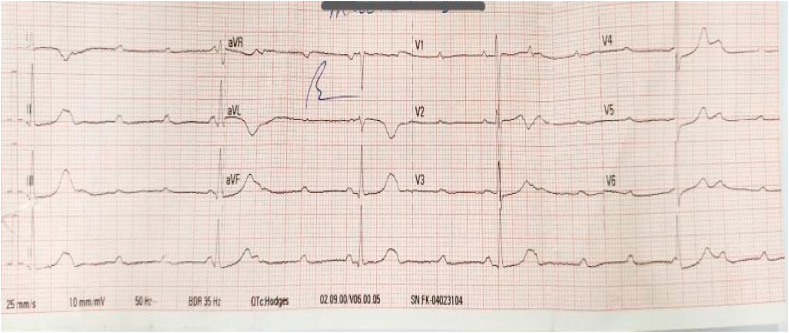
This is the electrocardiogram (ECG) of patient no 4 on her 3rd day of admission showing complete heart block.

Patient was given supplemental O_2_, and started on a course of IV Ceftriaxone 2 gm BID, Azithromycin 500 mg tablets BID, IV Clexane (1 mg/kg) BID, IV Methylprednisolone 1 g (05-2 mg/kg), Paracetamol 500 mg tablets TID, zinc and multivitamin supplements. On day 3 of hospitalization, her telemetry showed bradycardia to 40 bpm and ECG revealed evidence of complete heart block (Fig. 4). She had 2 syncopal episodes as well. Patient did not have any prior history of AV block or syncopal episode. Thorough history was taken from the patient to rule out any other cause of heart block including the absence of any AV nodal medication usage. Her serum potassium was 4.2 (3.5–5 mEq/L), there was no history of chest pain and her prior ECG did not show any evidence of recent onset ischemia, her thyroid function tests came back normal, her serial troponin were negative and TTE was unremarkable.

A TPM was inserted and her heart rate normalized to 70 bpm. She was then observed over a period of 5 days, during which she no longer had any further syncopal episodes and normal sinus rhythm was restored and her TPM was removed. Patient was discharged after making a full recovery from COVID-19. During her outpatient follow up, her repeat ECG all showed normal sinus rhythm with no evidence of CHB.

## 3 Discussion

As a result of a survey conducted by mainly electrophysiologists, it was determined that sinus bradycardia and CHB are the most common bradyarrhythmias that can occur in COVID-19 patients [8]. Diseases which affect the AV node conduction can occur in severely ill patients [9]. There are a number of mechanisms which can affect the AV conduction including metabolic disturbances, increase in vagal tone due to pain, manipulation of neck during nursing care and a variety of medications that affect the conduction system of the heart such as BB, CCB, and digoxin [10]. These variety of mechanisms can cause first degree, second degree and intermittent, but rarely can cause higher degree AV blocks [9]. Our cases had CHB which signifies etiology other than the ones described above in a critically ill patient.

The common etiologies of a complete heart block include ischemic heart disease, hyperkalemia, hypothyroidism, infections and rate limiting drugs affecting the AV node conduction including BB, CCB, and digoxin [11]. For all the patients discussed above, serial troponin markers were negative which ruled out ischemic heart disease, serum potassium levels were normal which rules out hyperkalemia, thyroid function tests were normal which eliminate hypothyroidism as a cause, medication history didn't reveal the use of rate limiting drug and patient did not have clinical features of any other infection other than the COVID-19. The occurrence of CHB in an otherwise healthy patient after the patient contracted COVID-19 infection makes the coincidence of CHB and COVID-19 infection very unlikely and it raises the speculation that both the diseases are related to each other. It was deemed that the underlying COVID-19 infection was the most likely culprit of CHB in our patients.

There are many different possible mechanisms that have been identified as to how qpatients affected by COVID-19 develop a CHB. These include hypoxemia, microthrombi, electrolyte disturbances, cytokine storm and direct invasion of cardiac myocytes and cardiac conduction tissues [8]. The exact mechanism which leads to the development of CHB in COVID-19 patients still remains unclear. There is also an ongoing debate regarding whether or not these cardiovascular effects will be temporary or permanent. Although it will require a prospective study to accurately determine potential residual cardiovascular damage caused by COVID-19, our case series have demonstrated the spontaneous resolution of AV block without the need of using a permanent pacemaker. This signifies that the cardiovascular effects of the novel virus are short lived and the affected patients will not suffer any long term cardiovascular outcomes.

### 3.1 Limitation

The limitation of this study was inability to rule out ischemia as a cause of complete heart block through angiography due to non-availability of the widespread angiographic facility as patients are covid-19 positive. Patients were not taken for MRI and other invasive procedures due to current active infection. We overcame this by searching for other surrogate markers of ischemia like ECG changes and cardiac markers.

## 4 Conclusion

The novel COVID-19 is continuing to evolve its clinical spectrum and can affect a wide variety of systems in the body including the cardiovascular system. This rare case series of occurrence of CHB in patients affected by COVID-19 will add more insight into the effects of this novel virus on the cardiovascular system. There has been much debate regarding the COVID-19's permanent effects on the cardiovascular system. This is going to require a prospective study to follow the evolution and the prognosis of different cardiovascular effects of the COVID-19 infection. Our case series showed that these effects are perhaps short-lived and will not have long-lasting effects. The goal of this case series is to show physicians that amongst the many potential effects of COVID-19, CHB should be considered and close monitoring with telemetry will be beneficial.

## Ethical approval

Obtained.

## Sources of funding

The authors declare no funding for this study.

## Author contribution

MHS, AWK, MK, wrote the abstract, case, study concept, design, conclusion; AH, VJ, DS reviewed paper, wrote discussion. MA, SA, TA, MH performed final edits Declaration of competing interest. The authors declare that they have no known competing financial interests or personal relationships that could have appeared to influence the work reported in this paper.

## Research registration number

1.Name of the registry: NA.

2.Unique Identifying number or registration ID: NA.

3.Hyperlink to your specific registration (must be publicly accessible and will be checked): NA.

## Guarantor

Talal Almas

RCSI University of Medicine and Health Sciences

123 St. Stephen's Green Dublin 2, Ireland

Talalamas.almas@gmail.com

+353834212442

## Provenance and peer review

Not commissioned, externally peer-reviewed.

## Declaration of competing interest

None.
